# Reactance to Social Authority in Entertainment-Education Media: Protocol for a Web-Based Randomized Controlled Trial

**DOI:** 10.2196/25343

**Published:** 2021-05-28

**Authors:** Alain Vandormael, Maya Adam, Violetta Hachaturyan, Merlin Greuel, Caterina Favaretti, Jennifer Gates, Till Baernighausen

**Affiliations:** 1 Heidelberg Institute of Global Health Heidelberg University Heidelberg Germany; 2 Department of Pediatrics Stanford University School of Medicine Stanford, CA United States; 3 Icahn School of Medicine, Mount Sinai New York, NY United States; 4 Africa Health Research Institute Durban South Africa; 5 Department of Global Health and Population Harvard T H Chan School of Public Health Boston, MA United States

**Keywords:** entertainment-education, sugar reduction, reactance, animated video, list experiment

## Abstract

**Background:**

Entertainment-education media can be an effective strategy for influencing health behaviors. To improve entertainment-education effectiveness, we seek to investigate whether the social authority of a person delivering a health message arouses the motivation to reject that message—a phenomenon known as reactance.

**Objective:**

In this study, using a short animated video, we aim to measure reactance to a sugar reduction message narrated by a child (low social authority), the child’s mother (equivalent social authority to the target audience), and a family physician (high social authority). The aims of the study are to determine the effect of the narrator’s perceived social authority on reactance to the sugar reduction message, establish the effectiveness of the video in improving behavioral intent to reduce the intake of added sugars, and quantify participants’ interest in watching the entertainment-education intervention video.

**Methods:**

This is a parallel group, randomized controlled trial comparing an intervention video narrated by a low, equivalent, or high social authority against a content placebo video and a placebo video. Using a web-based recruitment platform, we plan to enroll 4000 participants aged between 18 and 59 years who speak English and reside in the United Kingdom. The primary end points will include measures of the antecedents to reactance (proneness to reactance and threat level of the message), its components (anger and negative cognition), and attitudinal and behavioral intent toward sugar intake. We will measure behavioral intent using list experiments. Participants randomized to the placebo videos will be given a choice to watch one of the sugar-intervention videos at the end of the study to assess participant engagement with the entertainment-education video.

**Results:**

The study was approved by the ethics committee of Heidelberg University on March 18, 2020 (S-088/2020). Participant recruitment and data collection were completed in December 2020. The data analysis was completed in April 2021, and the final results are planned to be published by August 2021.

**Conclusions:**

In this trial, we will use several randomization procedures, list experimentation methods, and new web-based technologies to investigate the effect of perceived social authority on reactance to a message about reducing sugar intake. Our results will inform the design of future entertainment-education videos for public health promotion needs.

**Trial Registration:**

German Clinical Trials Registry DRKS00022340: https://www.drks.de/drks_web/navigate.do?navigationId=trial.HTML&TRIAL_ID=DRKS00022340.

**International Registered Report Identifier (IRRID):**

DERR1-10.2196/25343

## Introduction

### Background

Entertainment-education media can be an effective strategy for influencing health behaviors [1-3]. However, entertainment-education media face the same challenges as other traditional persuasion methods [4]. Persuasive health messages often fail to achieve the desired effect [5], and in some cases, may arouse the motivation to reject a message, a phenomenon known as reactance [6].

The theory of reactance comprises 4 elements [6]: (1) freedom, which individuals possess insofar as they are aware of it and can enact it; (2) threat to freedom, which involves any pressure on the individual, making it more difficult to enact that freedom; (3) reactance, which is the motivation to re-establish the freedom if that freedom is eliminated or threatened with elimination; and (4) direct restoration, which involves the freedom of the individual to perform a forbidden act. Research in this field has led to the development of several strategies to reduce reactance to health messages pertaining to littering [7], use of e-cigarettes [8], use of alcohol [9], and eating behaviors [10], among other health-related messages [11-16].

We are particularly interested in the effect of social authority on reactance to persuasive health messages. Our starting assumption is that an agent (human or otherwise) that delivers a health message possesses some influence or social authority [17]. For example, persons who have high social authority, such as experts or doctors, are often recruited to promote health messages [18-20]. However, research has shown that individuals may perceive health messages from experts as coercive, threatening, or having an ulterior motive [21], which could provoke reactance and negate the impact of the intervention [22,23].

### Objective

In a web-based entertainment-education video setting, there is limited, high-quality experimental evidence on the relationship between reactance and the perceived social authority of a message agent. Using a randomized controlled trial (RCT), we will evaluate the effect of social authority status on reactance to a short animated video on the intake of added sugars. The sugar message will be narrated by either (1) a preadolescent daughter, who has low authority status relative to the other narrators, (2) the daughter’s mother, who has equivalent social authority to the target audience, or (3) the family physician, who is an expert with high social authority. Results from this study will facilitate the development of videos for reducing reactance and improve the persuasiveness of health messages in web-based settings.

This study aims to achieve the following objectives:

1. Determine the effect of the narrator’s social authority (daughter, daughter’s mother, or family physician) on reactance to a sugar reduction message.
2. Establish the video’s effectiveness in improving behavioral intent to reduce the intake of added sugars.
3. Quantify participants’ interest in watching a short animated video about reducing the intake of added sugars.

Our null hypothesis is that the social authority of the child, who has low perceived social authority, the mother, who has equivalent perceived social authority, or the family physician, who has high perceived social authority, will have no effect on reactance to a video about reducing sugar intake.

## Methods

### Trial Design

This study consists of a parallel group RCT. Participants will be randomized to 1 of 5 arms: either the same sugar-intervention video narrated by a preadolescent daughter (arm 1: low social authority), the daughter’s mother (arm 2: equivalent social authority), or a family physician (arm 3: high social authority), or a content placebo video with a health message about tanning and sunscreen (arm 4: no sugar message), or a placebo video about earthquakes (arm 5: no sugar or health message). We will randomize the participants in a 1:1:1:1:1 ratio to the trial arms. Participants will watch 1 video once from start to finish.

Nested in each of the five trial arms is a list experiment. For each list experiment, participants will be randomized at a 1:1 ratio to a control or treatment group. The control group will receive a list of 5 items about behavioral intent (unrelated to sugar consumption). The treatment group will receive the same 5 items and a sensitive item about behavioral intent to reduce sugar intake. We will use the list experiment to reduce social desirability bias, as participants may already be primed to answer favorably to questions about sugar consumption.

**Figure 1:**
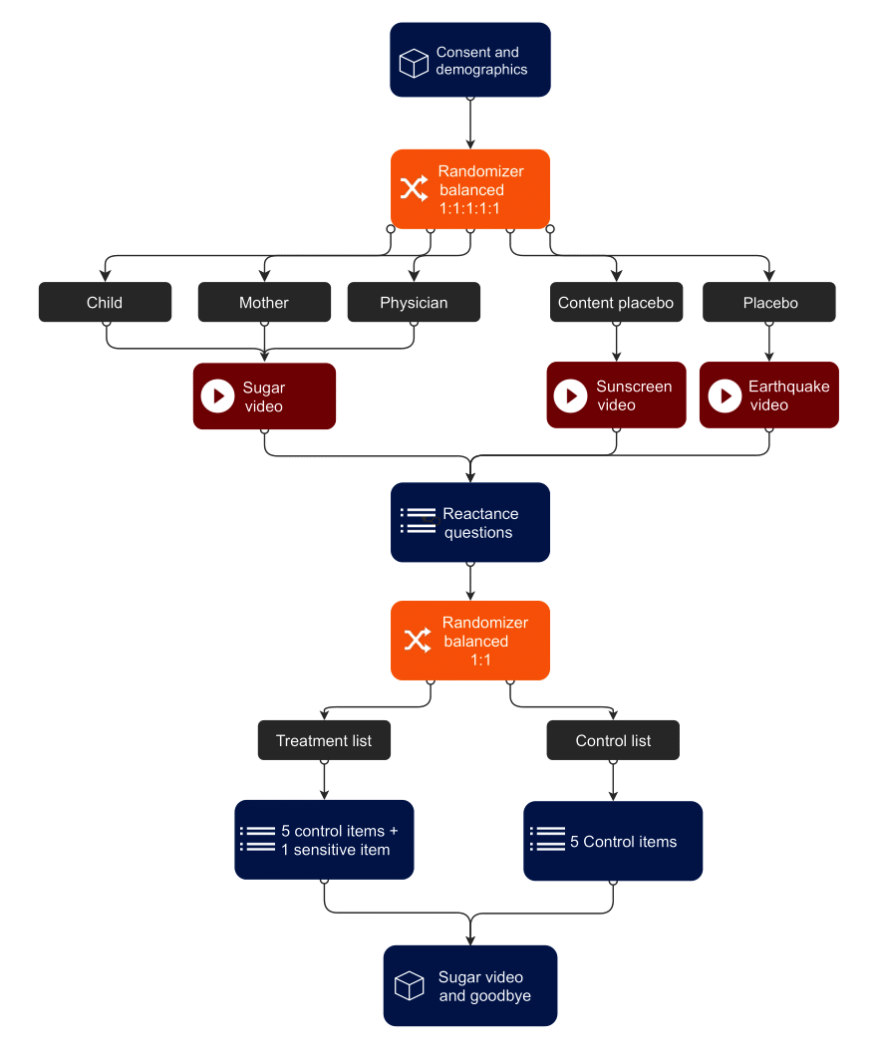
Trial design.

At the end of the study, participants assigned to the content placebo (*arm 4*) or placebo (*arm 5*) will be given a choice to watch the video intervention. Their choices will be recorded. Participants who choose to watch will be randomized at a 1:1:1 ratio to the sugar video narrated by the daughter, mother, or physician. The complete trial flowchart is presented in Figure 1.

### Study Setting

The study setting will be on the internet. We will use the web-based recruitment platform Prolific [24] to enroll study participants. We host and deploy our study on a web-based platform called Gorilla [25], a cloud platform that provides versatile tools for web-based, experimental, and behavioral research [26].

### Eligibility Criteria

Eligibility criteria included being between the ages of 18 and 59 years (male, female, or other), being able to speak English, and being a resident in the United Kingdom; not being eligible according to any of the inclusion criteria was the exclusion criteria. We will not exclude participants on an existing health condition (eg, diabetes) because Prolific does not collect health information from its users.

### Who Will Take Informed Consent?

The participants will undergo a process of informed consent. The consent form, which will be hosted on the Prolific platform, explains the purpose of the study, the risks and benefits of the research, and how a participant can contact the researcher or the human subjects review board at Heidelberg University. By clicking a link, participants will consent to participate in the study and will be redirected to the Gorilla platform. The Gorilla landing page contains additional information about the platform. Participants can exercise their freedom to not participate at any point during the study.

### Interventions

#### Intervention Description

The intervention consists of an entertainment-education video about reducing sugar intake [27-29]. Developed by our coauthor (MA) at the Stanford School of Medicine, the sugar video is animated and designed for a diverse and global audience. The 2 main characters, a mother and her preadolescent daughter, engage in food-related activities, such as grocery shopping and cooking dinner. The video presents educational content on health problems associated with consuming added sugars in foods, such as yogurt, chocolate milk, and breakfast cereals; a review of the World Health Organization recommendations for the daily consumption of added sugars is included. The narrative also includes the story of the father in this family, who dies from diabetes-associated complications because of frequent consumption of soda drinks. It concludes with a text message from the World Health Organization regarding the maximum number of teaspoons of sugar per day.

#### Explanation for the Choice of Comparators

We will compare the 3 intervention videos (*arms 1-3*) with each other (pairwise) to determine which social authority status is associated with the largest change in reactance and behavioral intent to reduce the intake of added sugars. In addition, we will compare the 3 intervention videos with the content placebo video (*arm 4*) and the placebo video (*arm 5*).

The content of the placebo video is similar in style to the sugar video. It is also animated, with a duration of 3.42 minutes, and has a health message about tanning and using sunscreen [30]. We used the content placebo video to isolate the *content effect* of the sugar-intervention video. It is possible that any video with a health message (eg, sunscreen protection) can improve overall health awareness and thus increase behavioral intent to reduce sugar intake. As both the intervention and content placebo videos have a health message, we expect that a significant difference in behavioral intent between the 2 videos (after random assignment) can be attributed to the content of the sugar message.

We will also compare each sugar-intervention video with a placebo video. The placebo describes the causes and characteristics of earthquakes [31] and contains no health-related or sugar consumption content. A significant difference in behavioral intent to reduce sugar intake between the content placebo and placebo videos (after random assignment) can therefore be attributed to the content of the sunscreen message. We call this difference the *health awareness effect*. We describe the *total intervention effect* as the difference between the sugar-intervention and the placebo videos, which is the sum of the content and health awareness effects.

We will also implement a list experiment in each arm with the control list as the comparator. The control list will include 5 items about general behavioral intent. The treatment list will include the same 5 control items plus a sixth item about behavioral intent toward reducing sugar intake. The control list (comparator) is needed to measure the prevalence of behavioral intent to reduce sugar intake, described in the *Behavioral Intent* section.

### Outcome Measures

#### Overview

**Figure 2:**
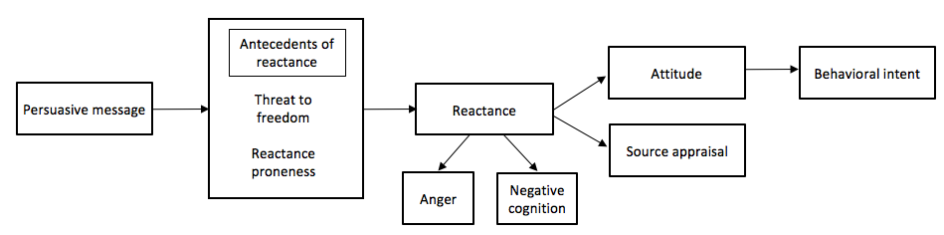
An intertwined process cognitive-affective model as described in Dillard and Shen with the addition of source appraisal from Zhang.

We will measure primary and secondary outcomes. Primary outcomes are based on the intertwined process cognitive-affective model, as described by Dillard and Shen [5] and Zhang [32] (Figure 2). In this model, there are two antecedents to reactance: the strength of the threat to freedom and trait proneness to reactance. Reactance is conceptualized as a mediator between the antecedents of reactance and behavioral intent to promote health-related activities. It is an intertwined process consisting of a cognitive and affective component that can be an experience of hostile, aggressive, or angry feelings. Furthermore, attitudinal and behavioral intentions are the consequences of reactance. The assessment of behavioral intentions can also help measure the direct restoration of freedom, which involves performing forbidden behavior and restoring participants’ need for self-determination and control [33]. In addition, source appraisal, which refers to the perception of the message source, is another important outcome of reactance [34].

#### Primary Outcome Measures

##### Trait Reactance Proneness

Trait reactance refers to reactance being a personal trait that causes some people to be more or less prone to experiencing reactance [35]. This implies that individuals tend to differ in their ways of perceiving and reacting to situations when their freedom is threatened, depending on their personalities. The propensity to trait reactance in this study will be measured using the Hong Psychological Reactance Scale developed by Hong et al [35]. The scale consists of 11 items that comprise 4 major factors: emotional response to restricted choice, reactance to compliance, resistance to influence from others, and reactance to advice and recommendations [35] (Textbox 1). These items are measured on a 5-point Likert scale with the following points: (1) strongly disagree, (2) disagree, (3) neither agree nor disagree, (4) agree, and (5) strongly agree [35].

**Trait reactance items based on the Hong Psychological Reactance Scale.**

**Emotional Response to Restricted Choice**

- I become frustrated when I am unable to make free and independent decisions.
- It irritates me when someone points out things that are obvious to me.
- I become angry when my freedom of choice is restricted.

**Reactance to Compliance**

- Regulations trigger a sense of resistance in me.
- I find contradicting others stimulating.
- When something is prohibited, I usually think, “That’s exactly what I am going to do.”

**Resistance to Influence From Others**

- I resist the attempts of others to influence me.
- It makes me angry when another person is held up as a role model for me to follow.
- When someone forces me to do something, I feel like doing the opposite.

**Reactance to Advice and Recommendations**

- I consider advice from others to be an intrusion.
- Advice and recommendations usually induce me to do just the opposite.

##### Threat to Freedom

To measure the threat level of the message, we will use the following 4 items from Dillard and Shen [5], each measured using a 5-point Likert scale:

1. The message threatened my freedom to choose.
2. The message tried to make a decision for me.
3. The message tried to manipulate me.
4. The message tried to pressure me.

##### Psychological Reactance

Following Dillard and Shen’s model, psychological reactance is assessed by measuring anger and negative cognition. Therefore, the average of all items on anger and negative cognition is an indicator of reactance. To measure anger, we will use a 5-point scale for the following 4 affirmations [5]:

1. This message makes me feel irritated.
2. This message makes me feel annoyed.
3. This message makes me feel aggravated.
4. This message makes me feel angry.

Negative cognition will be measured using the Likert scale from Quick et al [36-38] instead of the thought-listing procedure used by Dillard and Shen [5] because of the large sample size in this study. In a recent comparison of 3 methods for measuring negative cognition [39], the Likert scale was reported to have several advantages, including measurement economy as well as the flexibility to use these measures outside of the laboratory and when examining multiple candidate messages. The following 3 items, each measured on a 5-point Likert scale, will be used to measure negative cognition:

1. The thoughts I had while watching this video were mostly unfavorable.
2. The thoughts I had while watching this video were mostly negative.
3. The thoughts I had while watching this video were mostly bad.

##### Source Appraisal

Source appraisal will be assessed using the question, “The narrator of this video was...” and 7 semantic differential items anchored on either end with opposing adjectives: stupid or smart, unknowledgeable or knowledgeable, uninformed or informed, unintelligent or intelligent, unqualified or qualified, unreliable or reliable, and inexpert or expert [40]. The category ratings will be scored from 1 to 5, and higher scores will imply more unfavorable evaluations of the message source.

##### Attitude

Attitude toward message advocacy will be measured using four 5-point Likert items from Shen [41]:

1. I agree with what the message recommends.
2. I support what the message advocates.
3. I am in favor of the position in the message.
4. I endorse the claims made in the message.

Data from trait reactance proneness, threat to freedom, psychological reactance, source appraisal, and attitude will be used to assess objective 1.

##### Behavioral Intent

The behavioral intent to reduce the intake of added sugars will be measured using a list experiment approach. In Textbox 2, we present the 6 experiments and their list items. The control group will receive a list of 5 items, whereas the treatment group will receive the same list but with 1 additional sensitive item. The sensitive item covers the topics of natural versus added sugar, consumption of sugar-sweetened beverages, fresh fruit intake, reading of sugar content on package labels, teaspoons of sugar consumed per day, and home cooking.

We will assess whether participants are motivated (in lists 1, 3, and 4) or unmotivated (in lists 2, 5, and 6) to undertake the sensitive item. For example, in list 1, imagine that the control group selects an average of 2 out of the 5 items, and the participants in the treatment group select an average of 2.2 out of the 6 items. Holding all else equal, we conclude that the prevalence of participants who would cut their daily intake of sugar is 20%. The intention to restore one’s freedom will be present if there are higher scores for the *unmotivated* lists in the treatment group than the control group. The intention to reduce sugar intake will be present if there are higher scores for the *motivated* lists in the treatment group than the control group.

To avoid alerting the participant to the purpose of the list experiment and order effects, the 6 list experiments will be presented in random order. We designed the items to minimize ceiling and floor effects [42]. As described in the *Statistical Methods* section, we will use regression models to estimate the prevalence of each sensitive item [43]. These data will be used to assess objective 2.

**List experiment items. Each list experiment will be preceded by the question “How many of the five/six statements do you agree with? We don’t want to know which ones, just answer how many. This week I feel motivated/unmotivated to...”**

**List 1: Added Versus Natural Sugar**

- This week I feel motivated to...
  spend time watching TV.
  do the vacuuming in my home.
  spend time chatting with my friends on the web.
  pick a fight with my partner.
  rinse my nose with salt water daily.
  cut my daily intake of added sugar (sensitive item).

**List 2: Sugar-Sweetened Beverages**

- This week I feel unmotivated to...
  wash my hands frequently.
  spend time watching movies.
  clean the toilets in my home.
  smoke marijuana.
  clip my toenails.
  reduce the amount of sugar-sweetened beverages I drink (sensitive item).

**List 3: Fresh Fruit**

- This week I feel motivated to...
  open up a new savings plan at the bank.
  practice playing a musical instrument.
  watch a pornographic movie.
  do some shopping on the web.
  clean kitchen counters after use.
  eat fresh fruit daily (sensitive item).

**List 4: Food Labels**

- This week I feel motivated to...
  watch a new TV series.
  practice meditation daily.
  have alcoholic drinks on at least 3 evenings.
  catch up on last week’s work.
  clean all floor surfaces.
  check food labels for sugar content (sensitive item).

**List 5: Teaspoons of Sugar**

- This week I feel unmotivated to...
  clean my dishes after use.
  spend time on the internet.
  try learning a new language.
  play a prank on my partner.
  visit a car sales website.
  count how many teaspoons of added sugar I eat each day (sensitive item).

**List 6: Home Cooking**

- This week I feel unmotivated to...
  stock up on household supplies for a month.
  spend time gardening by myself.
  plan my next holiday.
  take a web-based course.
  go out with my friends.
  cook with fresh, whole foods (sensitive item).

#### Secondary Outcome Measure

We will measure participant engagement as a secondary outcome. At the end of the study, we will offer participants randomized to the placebo videos the choice to watch the sugar-intervention video or end the survey. The Gorilla platform will record this response. If the *Watch Video* button is clicked, Gorilla will randomize the participant to 1 of the 3 sugar videos and record the time (in milliseconds) from the start of the video until the participant clicks the *Finish* button or until the end of the video, whichever comes first. These data will be used to assess objective 3.

### Participant Timeline

**Figure 3:**
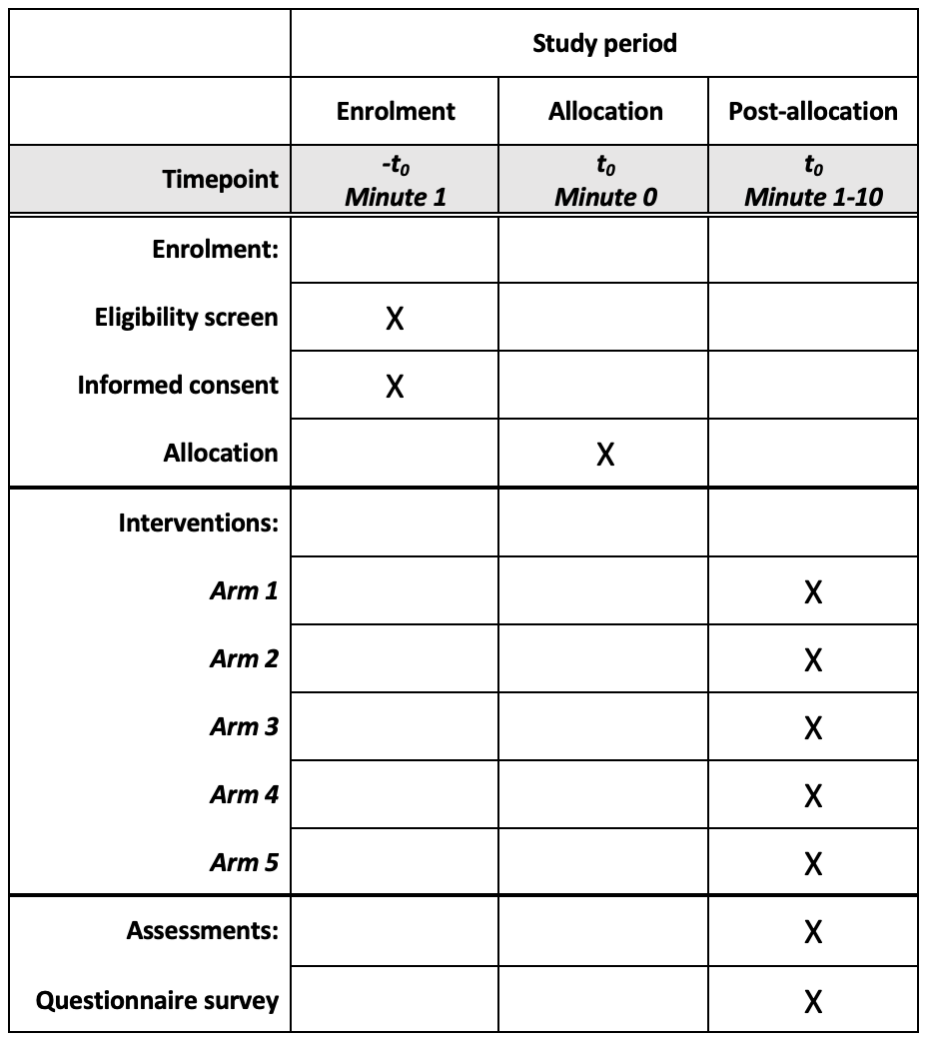
Schedule of enrollment, interventions, and assessments.

Participants are expected to finish the trial (watch the video, answer the survey questions, and complete the list experiment) in 10 minutes. The complete schedule of enrollment, interventions, and assessments is shown in Figure 3.

### Sample Size

We calculated the sample size needed for pairwise comparisons between the 3 groups using a one-way analysis of variance. The formula used to calculate the sample size is [44]


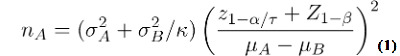


where *κ*=1, which is the matching ratio, *µ_A_* and *µ_B_* are the group A and B means, *σ_A_* and *σ_B_* are group A and B standard deviations, *α*=*.*05 is the type-1 error, β=*.*20 is the type-II error, *z* is the quantile function, and *τ*=2 is the number of comparisons to be made. For the control and treatment groups, we assumed a mean of *µ_A_*=2*.*0 and *µ_B_*=2*.*15, respectively (in other words, we expect, on average, that the control group will agree with 2 out of the 5 items and the treatment group will agree with 2.15% of the 6 items). We selected *σ_A_*=0*.*85 and *σ*=1*.*0; this calculation yields a sample size of n=769 per group. For a 5-way comparison, the sample size is n=3845. To ensure sufficient power and account for attrition, we will select a sample size of n=4000.

### Recruitment

We will use the Prolific platform to recruit the study participants. The user must create an account on Prolific and provide their personal information. Participants must agree with Prolific’s data privacy terms and conditions. Prolific will assign each participant a unique, anonymized ID. The study investigators will also create a Prolific account. We will instruct the Prolific platform on how many participants need to be recruited, and it will filter out all participants who do not meet the eligibility criteria. Participant entry into our study will happen on a *first-come, first -served* basis until the recruitment number (sample size) is reached. We will compensate the participants an equivalent of £1 (US $1.4) for the expected 10-minute completion time.

### Assignment of Interventions: Allocation

#### Sequence Generation

The Gorilla platform is specifically designed to host and implement web-based experimental studies. Gorilla will randomly allocate participants to the five trial arms.

#### Concealment Mechanism

The Gorilla platform uses a web-based randomization algorithm that is unknown to us.

#### Implementation

The Gorilla platform will complete the implementation.

### Assignment of Interventions: Blinding

#### Who Will Be Blinded?

As Prolific handles the interaction between the study investigators and participants, the participants will be completely anonymous to the study investigators. Only the participant’s unique, anonymized ID will be used to manage the linking between the Prolific and Gorilla platforms. The outcome measures will be self-reported and submitted anonymously. The study investigators and those involved in the data analyses and statistics will be blinded to the group allocation.

### Data Collection and Management

#### Plans for Assessment and Collection of Outcomes

Data will be collected on the Gorilla platform, where participants can submit data by clicking on the response buttons. We expect to collect data over a 1- to 2-week period.

#### Plans to Promote Participant Retention and Complete Follow-up

The expected completion time for the experiment is 10 minutes. Participants will be automatically timed out of Gorilla if they take longer than 45 minutes to complete the survey. The time-out is to ensure that participants do not clog up the system with incomplete surveys. As the participants will be anonymous to us, there is no way to initiate follow-up in the maximum 45-minute time limit.

### Data Management

All trial participants will be assigned a unique, anonymized string ID. The ID will be used on the Gorilla platform and linked to the participants’ responses. Gorilla will store the trial data on its cloud platform hosted on Microsoft Azure in the Republic of Ireland. The Gorilla database is encrypted using industry-standard cryptography. The study investigators own the research data collected using Gorilla and have complete control over it. The study investigators can generate and access the completely anonymized data from the Gorilla platform. The data will be downloaded and stored safely for statistical analysis on a computing system maintained by Heidelberg University in Germany.

### Confidentiality

Participants, who are completely anonymous to us, will have no identifying information associated with their unique IDs. We will inform participants that if they email the study investigators then their names could be revealed to us. The study investigators will keep this information confidential.

### Statistical Methods

#### Statistical Methods for Primary and Secondary Outcomes

For the descriptive statistics, we will obtain means and SDs of age, sex, country of residence, and education status variables. We will use an analysis of variance to estimate pairwise differences in means between the sugar-intervention videos, content placebo video, and placebo video. We will use the Tukey range method to create confidence intervals for all pairwise differences between the means while controlling for the family error rate. Within each trial arm, we will estimate the prevalence of behavioral intent as the difference between the treatment and control list for each list experiment. From these estimates, we will obtain the differences in means between trial arms. This approach is analogous to a difference-in-difference analysis, which we will implement by specifying the main and interaction terms in an ordinary least squares (OLS) regression model. The OLS equation for each list experiment is given as follows:

y = b_0_ + b_1_*VideoArm* + b_2_*TreatList* + b_3_*VideoArm* × *TreatList*

where y is the number of statements in the list that the participant agreed with, *VideoArm* indicates the arm to which the participant was assigned, and *TreatList* indicates if the participant was assigned to the treatment list within that arm. We will calculate standard errors, 95% CIs, and *P* values for linear combinations of coefficients from the OLS model. We will use R statistical software to perform the analysis.

#### Methods in Analysis to Handle Protocol Nonadherence and Any Statistical Methods to Handle Missing Data

Participants who do not complete the survey will be excluded from the final analysis. This loss will be reported.

### Plans for Granting Public Access to the Full Protocol, Participant-Level Data, and Statistical Code

This document is the full protocol. Additional data or documentation can be requested from the corresponding author.

### Oversight and Monitoring

#### Composition of the Data Monitoring Committee, Its Role, and Reporting Structure

As the intervention is relatively short and takes place on the web, a data monitoring committee is not needed.

#### Adverse Event Reporting and Harms

As participants are anonymous to us, we will not be able to report any adverse events or harm. It is unlikely that there will be adverse events, given the format of our 10-minute web-based trial.

#### Criteria for Discontinuing or Modifying Allocated Interventions

We will not discontinue or modify the allocated interventions during the course of the study.

#### Provisions for Posttrial Care

After completing the study, participants in the health awareness placebo and content placebo arms will receive the sugar video as postaccess to treatment.

#### Plans for Communicating Important Protocol Amendments to Relevant Parties

All relevant parties, including the ethics committee of the University of Heidelberg and the German Clinical Trials Register, will be notified about any modifications to the protocol that may impact the conduct of the study, the potential benefit of participants, or participant safety.

#### Dissemination Plans

We will disseminate the study findings through journal publications and conference presentations.

### Ethical Approval and Consent to Participate

Ethical approval was obtained from the Heidelberg University’s ethics committee (Universität Heidelberg Ethikkommission der Medizinische Fakultät) on March 18, 2020, protocol S-088/2020. All participants will undergo a process of informed consent. The consent form, which will be hosted on the Prolific platform, explains the purpose of the study, the risks and benefits of the research, and how a participant can contact a researcher (and/or the human subjects review board at Heidelberg University). By clicking the link, participants consent to participate in the study and are redirected to the Gorilla platform. The landing page contains additional information about the Gorilla platform. Participants can exercise their freedom to not participate at any point during the study (see Multimedia Appendix 1 for the informed consent form).

### Availability of Data and Materials

The data will be collected and stored on the Gorilla platform. The study investigators own and have complete control of the research data, which can be accessed at any time. For statistical analysis, the data will be downloaded and safely stored in a computing system maintained by the University of Heidelberg.

## Results

The study was approved by the Heidelberg University ethics committee on March 18, 2020 (S-088/2020). Participant recruitment and data collection were completed in December 2020. The data analysis was completed in April 2021, and the final results are planned to be published by August 2021.

## Discussion

### Principal Findings

There is growing evidence that entertainment-education media can be an effective strategy for promoting healthy behaviors [45-48]. However, further research is needed to understand which entertainment-education components can be modified to reduce reactance to health messages [3,49,50]. In this proposed study, we focus on a modifiable component—the perceived social authority of the health messenger—and its effect on reactance to a message about reducing sugar intake.

In recent years, video-based animation has emerged as a potentially powerful entertainment-education strategy for changing behavior [51-53]. This animation format has enabled the creative use of nonhuman and nonadult characters to promote more persuasive health messages [54]. We leveraged this animated format to create 3 culturally neutral characters that narrate a health message about sugar consumption. During the design phase, we assumed that a child narrator would be a more persuasive messenger because she would be perceived as nonthreatening or lacking a vested interest and therefore would be less likely to arouse reactance when compared with an adult narrator. However, we also considered that the child would not be taken seriously or that her lack of expertise would nullify the persuasiveness of the health message. We were unable to find prior research studies to inform our decision to use a child narrator. To this end, we propose an RCT to investigate whether reactance to the sugar message would be reduced if it was narrated by a preadolescent daughter, the daughter’s mother, or a family physician. Our results may show that a child can be a powerful and persuasive health promotion agent, which could inform future choices regarding the design and delivery of health messages.

### Strengths and Limitations

In a systematic review, Shen and Han [4] concluded that there is a lack of experimental methods to evaluate the effectiveness of entertainment-education media. They call for “controlled experiments to uncover the cognitive and/or affective factors that mediate entertainment-education’s effects” [4]. Our protocol responds to this call by leveraging experimental methods and carefully considering several factors that may mediate the role of social authority on reactance to the sugar reduction message, which we discuss below.

First, our study will use an RCT design to randomize participants to 1 of 3 sugar-intervention videos, a content placebo video, or a placebo video. The 3 intervention videos are exactly the same, except that the sugar message is narrated by the daughter, the daughter’s mother, or the family physician. The RCT should ensure that the enrollment stage does not introduce systematic differences between trial arms. For example, participants may have pre-existing health conditions, such as diabetes, which could affect their responses to the survey questions. However, random assignment will take care of this potential source of bias by distributing it uniformly across trial arms. Thus, holding all else equal, and because of randomization, differences in reactance toward the sugar message should be because of the experimental manipulation of the narrator’s social authority.

Second, the content placebo and placebo videos are an innovative feature of our study and will enable us to isolate the health awareness effect and content effect of the intervention video. The content effect can be quantified as the difference in mean state reactance (MSR) between the intervention arm and the content placebo arm. As both videos promote a health message, and because we will randomize, any significant difference in MSR should be because of the sugar reduction content of the intervention video. We can calculate the health awareness effect as the difference in MSR between the content placebo arm and placebo arm. As the content placebo video promotes a health message and the placebo video does not, the difference in MSR should be because of the health awareness generated by the sunscreen message. Thus, we can decompose the total intervention effect—the difference in MSR between the intervention and placebo arms—as the sum of health awareness and content effects. We are not aware of any previous entertainment-education study that has used an experimental approach to partition the effect of an intervention video in this way.

We do not believe that the differences between the placebo videos (which we did not create) and the sugar-intervention videos (which we created) will confound our results. Such differences may relate to the animation style, background shapes or colors, and target audience, among other design decisions. As mentioned, the main interest of our study is the difference in state reactance toward the sugar reduction message following random assignment to the 3 social authority levels. On the basis of the theoretical model described earlier, it is only the content of the message or the characteristics of the messenger that can threaten an individual’s freedom and arouse reactance. As the placebo video of earthquakes does not promote a health message, we expect it to arouse a very small (or even null) level of state reactance (the type of animation style of the placebo videos, for example, cannot realistically threaten an individual’s *freedom*). Therefore, the placebo video will provide a baseline measure of state reactance, which will enable us to quantify the content and health awareness effects. Further, we were very careful to select a content placebo video in which the messenger had culturally neutral or agnostic characteristics. In this case, the narrator of the sunscreen message is not seen, and it is not possible to determine his social authority. A possible exception is that the placebo videos are narrated by male voices and the sugar-intervention videos by female voices. However, it is unlikely that this design difference will be sufficiently large to bias our results significantly.

Third, we will conduct a list experiment in each of the 5 arms. This is the second experimental method that we leverage in our study design. We use a list experiment to reduce social desirability bias in participants’ responses to the behavioral intent questions about reducing sugar intake.It is likely that participants will already be primed to give socially acceptable responses to questions about their health and sugar consumption. The indirect questions (ie, how many statements do you agree with) provide protection to participants if they want to reject the sugar message without revealing this intention. To the best of our knowledge, few (if any) studies have used a list experiment approach to evaluate the effectiveness of an entertainment-education video to improve a given health outcome.

Finally, we will also use an experimental approach to measure participants’ engagement with the sugar videos. We will do this by *piggy-backing* this objective on the ethical requirement to give participants not randomized to the intervention the opportunity of watching the intervention video at the end of the study. Participants will be informed that they will not be compensated for their additional time, thus enabling us to estimate the proportion of participants who will voluntarily watch the sugar video and how long they watch the video. These findings will help us determine participants’ willingness to watch an entertainment-education video, especially when this willingness must be balanced against a time cost, as is generally the case in a real-world scenario.

### Conclusions

We expect that our study will make important contributions to entertainment-education literature. The lessons learned can help us improve the design of entertainment-education videos that facilitate disseminating persuasive health messages to a global audience at a rapid scale.

## Appendix

Multimedia Appendix 1 Consent form provided to participants.

## Abbreviations

MSR: mean state reactance
OLS: ordinary least squares
RCT: randomized controlled trial
