# Supplementary material for: Reactance to Social Authority in Entertainment-Education Media: Protocol for a Web-Based Randomized Controlled Trial
Source: JMIR Res Protoc. 2021 May 28;10(5):e25343. doi: 10.2196/25343 (PMC8196361; doi:10.2196/25343)
Supplement: Multimedia Appendix 1 [file resprot_v10i5e25343_app1.pdf]

Information and Consent Form

Dear Participant,

You are being invited to participate in a research study titled *"A study to measure participant responses to a short, animated informational video."* This study is being done by Dr. Alain Vandormael, Dr. Till Bärnighausen, and Dr. Maya Adams from the University of Heidelberg at:

Heidelberg Institute of Global Health,
130.3 Im Neuenheimer Feld,
Heidelberg, Germany
69120

Why are we doing this research study? The purpose of this research study is to evaluate the effectiveness of entertainment-education video on health.

Who can participate in this research study? Any person who has registered on the Prolific. You are eligible to participate in this study if you can speak English, are between the ages of 18 and 59, and currently reside in the United States or the United Kingdom.

What will I be asked to do and how much time will it take? If you agree to take part in this study, you will watch a video and be asked to complete an online survey. This survey will ask about your age, sex, educational background, and various questions that may or may not be related to the video. The video and the survey will take you approximately *10* minutes to complete.

Will being in this research study help me in any way? You may not directly benefit from this research; however, we hope that your participation in the study may improve your knowledge on the topic.

What are my risks of being in this research study? We believe there are minimal risks associated with this research study; however, a risk of breach of confidentiality always exists and we have taken the steps to minimize this risk as outlined in a section below.

Who can I talk to if I have questions? If you have questions about this project or if you have a research-related problem, you may contact the researcher(s), Dr Alain Vandormael at alain.vandormael@uni-heidelberg via the Prolific platform. The messaging system on Prolific protects your anonymity and we will not be able to access your personal information (name, email address) in any way.

How will my personal information be protected? We will not ask you for any personal information. You will be completely anonymous to us. All your answers in this study will remain confidential. Your Prolific ID will be stripped from the data and NOT be shared with anyone. The stripped data may be seen by a limited number of researchers involved in the study. All information collected for this study will be securely kept and only accessed by authorized research team members.

What happens if I say yes, but I change my mind later? You do not have to be in this study if you do not want to. If you agree to be in the study, but later change your mind, you may drop out at any time for any reason. There are no penalties or consequences of any kind if you decide that you do not want to participate. However, you will not be rewarded for an incomplete survey.

Will I be given any money or other compensation for being in this research study? You will receive a reward of £1.00 for the expected 10-minute completion time.

This research has been reviewed by the Ethics Committee at the Faculty of Medicine, Heidelberg University. If you wish to speak with someone from the Ethics Committee, you may contact +062 21 562 6460 or email ethikkommission-I@med.uni-heidelberg.de

By clicking “I agree” below you are indicating that you are at least 18 years old, have read this consent form and agree to participate in this research study.

Please print a copy of this page for your records.

☐ I understand and want to participate in this study.

My check mark indicates that the information in the consent document and any other written information was accurately explained to, and apparently understood by, the participant or the participant’s legally authorized representative, and that informed consent was freely given by the participant or the legally authorized representative.
